# Supplementary material for: The Neurospora photoreceptor VIVID exerts negative and positive control on light sensing to achieve adaptation
Source: Mol Syst Biol. 2013 May 28;9:667. doi: 10.1038/msb.2013.24 (PMC4039372; doi:10.1038/msb.2013.24)
Supplement: Supplementary information — Supplementary Figures S1–3, Supplementary Tables S1–2 [file msb201324-s1.pdf]

Supplementary Information

The Neurospora photoreceptor VIVID exerts negative and positive control on light sensing to achieve adaptation

Elan Gin, Axel C.R. Diernfellner, Michael Brunner, Thomas Höfer

Table of Contents

Figure S1. Dilution series to test the probe efficiencies.
Figure S2. VVD stabilizes WCC protein levels
Figure S3. Transfer from light to dark results in the onset of oscillations.
Text S1. Mathematical Model
Text S2. Futile cycling provides the means for repeated induction
References

MATLB code for all model simulations are available as separate supplementary files.

Supplementary Figures

Figure S1

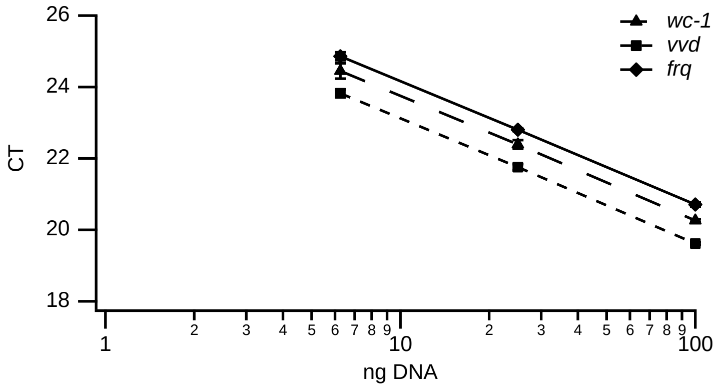

Figure S1: **Dilution series to test the probe efficiencies.**
To ascertain the relative differences between *vvd*, *wc-1* and *frq* RNA levels and thus be able to compare them with each other, we tested the efficiency of their respective primers and probes in a qRT-PCR using a dilution series of wildtype gDNA as template. Calculating the delta CT with *vvd* at the lowest DNA template concentration as reference, we determined the fold difference in the probe efficiencies: *vvd* = 1; *frq* = 2.086 and *wc-1* = 1.552. The values of the experiment (Fig. 1) were then corrected accordingly, with reference to *vvd*. qRT-PCR with a dilution series (6.25, 25 and 100 ng) of wild type gDNA as template probing for *frq* (diamonds), *wc-1* (triangles) and *vvd* (squares). Threshold cycles (CT) are plotted against the amount of DNA in the samples (n=3; ±SD)

Figure S2

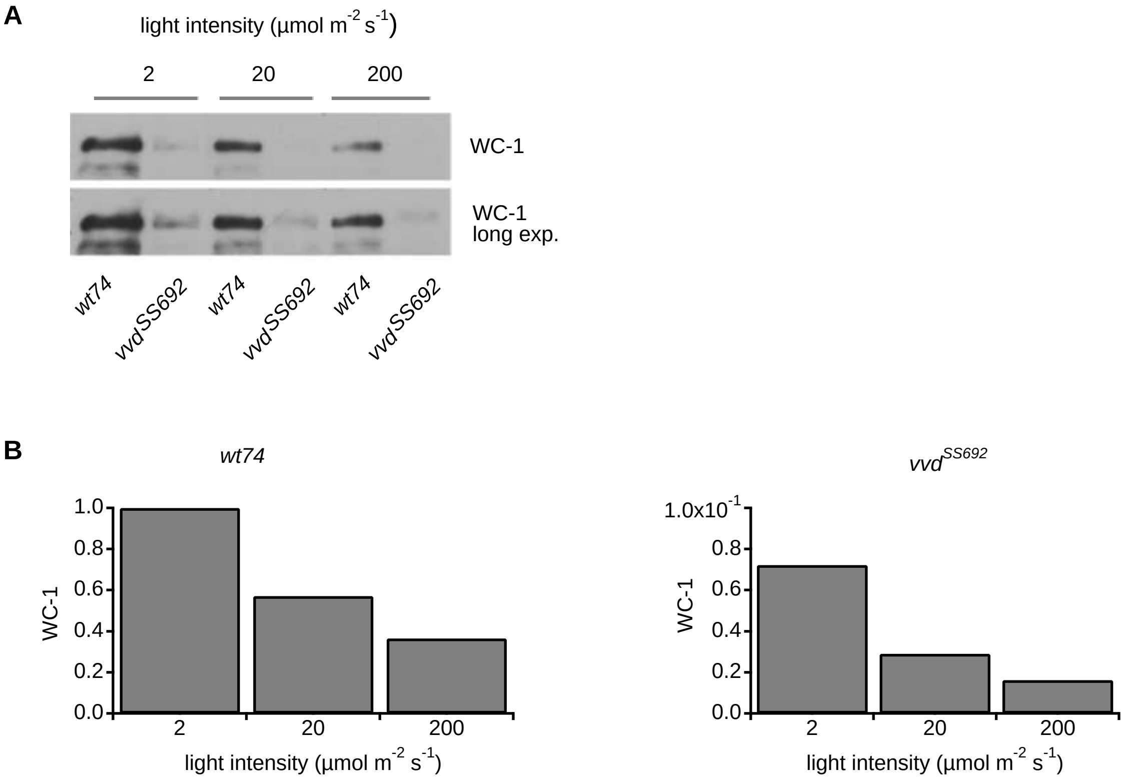

Figure S2: **VVD stabilizes WCC protein levels**
(A) WC-1 protein levels depend on light intensity. Mycelia from *wt74* and vvd^{SS692} were grown for 48 hours at the indicated light intensities and western blots were probed for WC-1. Short and long exposures are shown. (B) Western blots were quantified showing that WC-1 levels in the vvd^{SS692} strain are approximately one-tenth of the wildtype levels. Note the scale on the vertical axes.

Figure S3

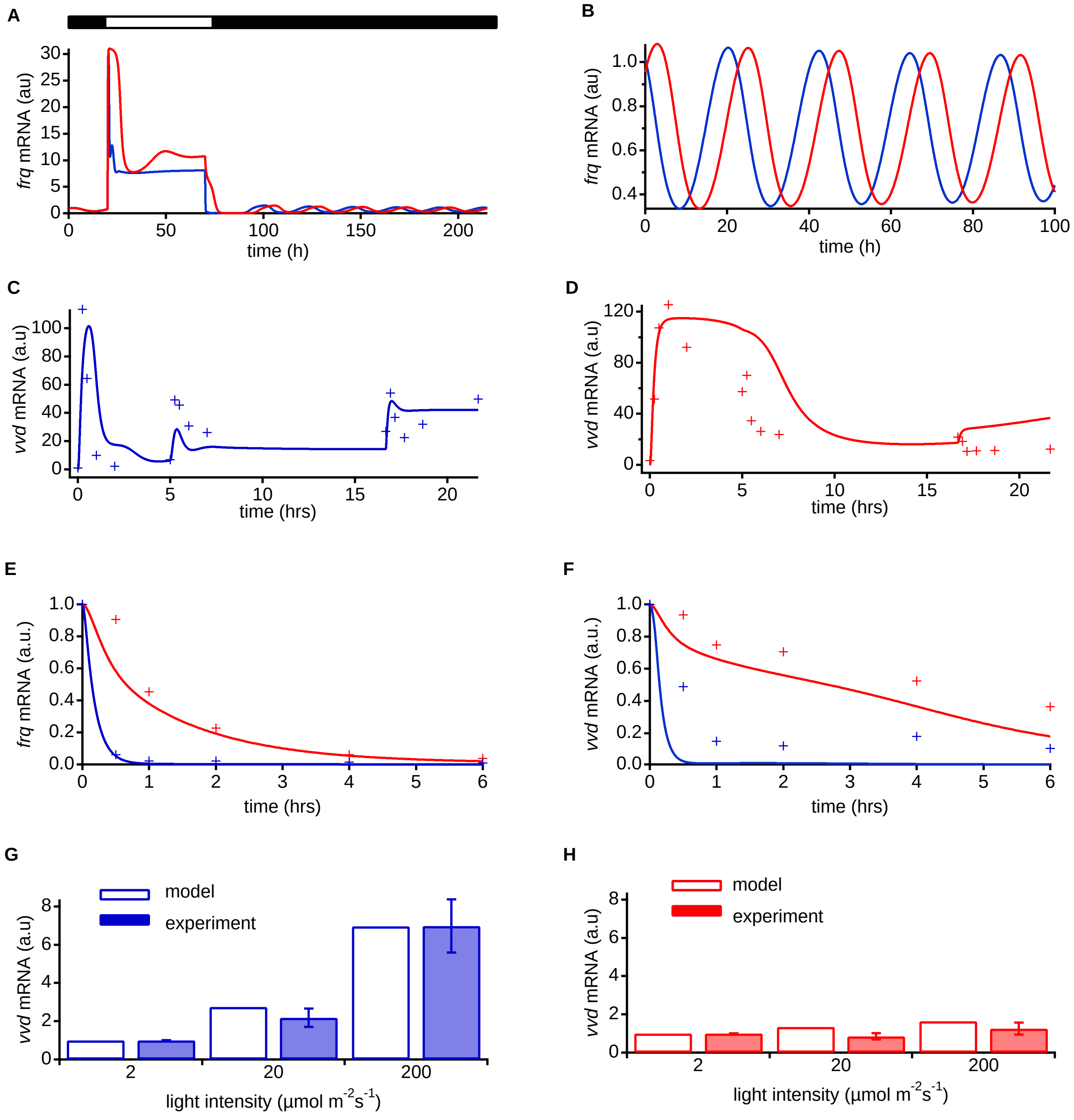

Figure S3: **Transfer from light to dark results in the onset of oscillations.** Continued on the following page.

Figure S3: **Transfer from light to dark results in the onset of oscillations.**
Self-sustained oscillations in constant darkness are thought to arise through delayed negative feedback of the WCC target FRQ on the activity of the WCC (Brunner and Káldi, 2008; Baker *et al*, 2012). These delays are included in the form of a series of intermediate steps between FRQ synthesis and FRQ-dependent inhibition of the WCC:
frq mRNA $\xrightarrow{k_{15}}$ FRQ_1 $\xrightarrow{k_{p1}}$ FRQ_2 $\xrightarrow{k_{p2}}$ FRQ_n $\xrightarrow{k_{pn}}$ FRQ $\xrightarrow{k_{19}}$ $\emptyset$,
where the final FRQ mediates phosphorylation of the WCC. The additional parameters are: k_{p1} = 0.375 hr^{-1}; k_{p2} = 0.375 hr^{-1}; k_{pn} = 0.375 hr^{-1} A small set of modifications had to be made to the other parameters to ensure that adaptation to light is retained. Modified parameter values: k_3=10.0 hr^{-1}; K_3 = 6.0 au; n_3 = 5.0; K_4 = 61.0 au; k_{-4} = 0.3 hr^{-1}; k_{11} = 43.0 hr^{-1}; K_{11} = 0.07 au; k_{19} = 0.24 hr^{-1}; q_6 = 750.0 hr^{-1}; Q_6 = 0.07 au. The modification of parameter values and the introduction of time-delayed FRQ kinetics do not affect the adaptation behavior of the system. In all cases, the blue curves correspond to the wild type, the red curves to the *vvd-* mutant. Solid curves represent the model simulation results; dashed lines, the experimental results.
(A) Simulation of light induction of *frq* mRNA followed by transfer to darkness shows the characteristic adaptation to light before the onset of oscillations in the dark. In the *vvd-*, *frq* mRNA levels decrease slower than in the wild type after the transfer from light to dark (cf Figure 6) resulting in a delay in onset of oscillations. Apart from this, the period and amplitude of the oscillations are the same between the wild type and the *vvd-* mutant, confirming that oscillations in free-running conditions are independent of VVD.
(B) *frq* mRNA levels oscillate in the dark with oscillations phase-shifted in the *vvd-* by approximately 4 hours, an observation that has previously been noted (Heintzen *et al*, 2001; Elvin *et al*, 2005; Hunt *et al*, 2007).
(C)-(D) Responsiveness and adaptation to increasing light steps show that the wild type remains responsive and adapts within 4 hours, while the *vvd-* mutant requires longer for adaptation to be achieved.
(E)-(F) A light to dark transfer shows that transcriptional activity for *vvd* and *frq* mRNA are unregulated for up to 4 hours in the *vvd-* mutant, while activity drops within 1 hour in the wild type.
(G)-(H) Steady-state *vvd* mRNA levels are shown experimentally to increase with increasing light intensity, while the levels remain constant in the *vvd-* mutant. Model simulations agree with the experimental results.

Supplementary Text 1

Mathematical model

Based on time-resolved experimental data we constructed a mathematical model to understand the role of VVD as both a negative and positive regulator in light adaptation.

We outline below, some of the model features describing the interaction between the circadian transcription factor the White Collar Complex (WCC) and its negative regulators VIVD (VVD) and Frequency (FRQ). Saturation terms have been used for enzymatic reactions: induction of gene expression and phosphorylation steps by FRQ. Photoactivation, complex formation, dissociation and (non-enzymatic) photoadduct have been modeled by mass action terms.

1. **Light-activation of WCC and VVD**

   WC-1 and VVD contain a flavin-binding light-oxygen-voltage (LOV) domain, the blue-light sensors in the light receptors of plants and fungi. Light triggers a LOV-mediated homodimerization of the WCC, which induces expression of a large number of genes.

   We model the light activation of both the WCC and VVD by a two-step process describing first activation by light (to give the light-activated form of the WCC, WCC*) followed by homodimerizaton of WCC*. Both steps are modeled using mass action kinetics.

   The effect of light is modeled through a direct change to the activation rates of the dark forms of unphosphorylated and phosphorylated WCC (WCC, $\mathrm{WCC_p}$), k_1, and VVD (VVD), q_1, to form the corresponding light-activated forms, WCC*, $\mathrm{WCC^*_p}$ and VVD*.

   Values for the activation rate are estimated from Malzahn *et al*, 2010 and given in Table S1.

Light intensity	Value (k_1, q_1)
2 μmol $\mathrm{m^{-2}s^{-1}}$	1.2 $\mathrm{hr^{-1}}$
20 μmol $\mathrm{m^{-2}s^{-1}}$	5 $\mathrm{hr^{-1}}$
200 μmol $\mathrm{m^{-2}s^{-1}}$	40 $\mathrm{hr^{-1}}$

Table S1: Light-activation rates, k_1, q_1, for each of the experimental light intensities used.

2. **Transcription**

   The WCC drives transcription of many genes, including *vvd*, *frq* and *wc-1*.

   In the dark, the inactive, dark form of the WCC can drive expression of *frq* by binding to the clock-box (C-box) element of the *frq* promoter (Froehlich *et al*, 2003). This is given by the second term in the equation for $\frac{d[\mathrm{FRQ}]}{dt}$, where WCC binds with affinity K_{14}. Additionally, light-activated WCC (in the form of the homodimer, WCC*-WCC*) can also bind to the C-box and to the proximal light-responsive element (LRE) of *frq*. Binding affinity of WCC*-WCC* to the C-box is given by $K_{14'}$, while WCC*-WCC* binds to the LRE with affinity K_{13}.

   vvd transcription is driven only by the light-activated homodimer (WCC*-WCC*) with a maximal transcription rate of q_6.

   Transcription of *wc-1* in the dark is independent of the WCC, given by the basal rate k_{12}. In light, transcription of the WCC is driven by the homodimer.

3. **WCC-VVD heterodimerization**

   As suggested by previous experimental works, the VVD protein is key to the light regulation process through the competitive formation of WCC-VVD heterodimers through interaction of their LOV domains (Chen *et al*, 2010; Malzahn *et al*, 2010). In the model, we assume that the light-active forms of the WCC and VVD (WCC* and VVD*) can form a heterodimer (WCC*VVD*) and that the light-active, phosphorylated WCC ($\mathrm{WCC^*_p}$ and VVD*) can also form a heterodimer ($\mathrm{WCC^*_pVVD^*}$).

The lifetime of the heterodimer is governed by: (1) degradation of the complex (l_2, l_3), (2) dissociation of the complex (l_{-1}) and (3) degradation of the photoadduct, with the WCC* or VVD* component reverting to their dark form. For (3), the following combinations can occur:

- WCC*-VVD* $\xrightarrow{l_4}$ WCC* + VVD
- WCC*-VVD* $\xrightarrow{l_5}$ WCC + VVD*
- WCC_p^*-VVD* $\xrightarrow{l_6}$ WCC_p^* + VVD
- WCC_p^*-VVD* $\xrightarrow{l_7}$ WCC + VVD*

4. **VVD homodimerization**

   VVD dimerizes in a light-dependent manner, forming a rapidly exchanging equilibrium between the monomer and the dimer (Zoltowski and Crane, 2008). Dimerization of VVD competes with heterodimerization, thereby allowing formation of the transcriptionally-active WCC homodimer. We model this in the same way as light-activation of the WCC and homodimerization of the activated WCC, by a two-step process that involves first activation of the VVD protein and then homodimerization of the activated species.

5. **FRQ-mediated phosphorylation of the WCC**

   The WCC-FRQ negative feedback loop forms the core of the circadian clock. Phosphorylation of the WCC by FRQ inactivates the WCC and therefore, its own transcription.

   Experimental evidence has shown that the FRQ level is much lower than that of the WCC (Schafmeier *et al*, 2005, 2006) leading to the conclusion that FRQ modulates phosphorylation of WCC, rather than binding to WCC in a 1:1 stoichiometry.

Model equations

Based on the above details, we developed a system of 14 ordinary differential equations describing changes in protein and mRNA concentrations of WCC, VVD and FRQ. The variables are listed in Table S2. Note that we denote WCC by W and VVD by V. An asterix indicates the light-activated form of the protein. *wcc*, *vvd* and *frq* mRNA concentrations are denoted by mWCC, mVVD and mFRQ, respectively.

Model variables	
inactive (dark form) WCC protein	W
light-activated WCC protein	W^*
WCC-WCC protein homodimer	W^*W^*
inactive phosphorylated WCC protein	W_p
light-activated phosphorylated WCC protein	W_p^*
inactive VVD protein	V
light-activated VVD protein	V^*
VVD-VVD protein homodimer	V^*V^*
WCC-VVD heterodimer	W^*V^*
phosphorylated WCC-VVD heterodimer	$W_p^*V^*$
FRQ protein	FRQ
wcc mRNA	mWCC
vvd mRNA	mVVD
frq mRNA	mFRQ

Table S2: Model variables.

$$
\begin{aligned}
\frac{d[\mathrm{W}]}{dt} &= -k_1[\mathrm{W}] + k_{-1}[\mathrm{W}^*] + 2l_8[\mathrm{W}^*\mathrm{W}^*] + k_{-3}[\mathrm{W_p}] - [\mathrm{W}]\frac{k_3[\mathrm{FRQ}]^{n_3}}{[\mathrm{FRQ}]^{n_3} + K_3^{n_3}} + \frac{k_{16}[\mathrm{mWCC}]^{n_{16}}}{K_{16}^{n_{16}} + [\mathrm{mWCC}]^{n_{16}}} \\
&- k_6[\mathrm{W}] + l_5[\mathrm{W}^*\mathrm{V}^*], \\
\frac{d[\mathrm{W}^*]}{dt} &= k_1[\mathrm{W}] - k_{-1}[\mathrm{W}^*] + 2k_{-2}[\mathrm{W}^*\mathrm{W}^*] - 2k_2[\mathrm{W}^*]^2 - [\mathrm{W}^*]\frac{k_4[\mathrm{FRQ}]^{n_4}}{([\mathrm{FRQ}]^{n_4} + K_4^{n_4})} + k_{-4}[\mathrm{W_p^*}] \\
&+ -k_7[\mathrm{W}^*] + l_{-1}[\mathrm{W}^*\mathrm{V}^*] - l_1[\mathrm{W}^*][\mathrm{V}^*] + l_4[\mathrm{W}^*\mathrm{V}^*], \\
\frac{d[\mathrm{W}^*\mathrm{W}^*]}{dt} &= k_2[\mathrm{W}^*]^2 - k_{-2}[\mathrm{W}^*\mathrm{W}^*] - l_8[\mathrm{W}^*\mathrm{W}^*] - k_8[\mathrm{W}^*\mathrm{W}^*], \\
\frac{d[\mathrm{W_p}]}{dt} &= -k_1[\mathrm{W_p}] + k_{-1}[\mathrm{W_p^*}] - k_{-3}[\mathrm{W_p}] + [\mathrm{W}]\frac{k_3[\mathrm{FRQ}]^{n_3}}{[\mathrm{FRQ}]^{n_3} + K_3^{n_3}} - k_9[\mathrm{W_p}] + l_7[\mathrm{W_p^*}\mathrm{V}^*], \\
\frac{d[\mathrm{W_p^*}]}{dt} &= +k_1[\mathrm{W_p}] - k_{-1}[\mathrm{W_p^*}] + [\mathrm{W}^*]\frac{k_4[\mathrm{FRQ}]^{n_4}}{([\mathrm{FRQ}]^{n_4} + K_4^{n_4})} - k_{-4}[\mathrm{W_p^*}] - k_{10}[\mathrm{W_p^*}] + l_{-1}[\mathrm{W_p^*}\mathrm{V}^*] - l_1[\mathrm{W_p^*}][\mathrm{V}^*] \\
&+ l_6[\mathrm{W_p^*}\mathrm{V}^*], \\
\frac{d[\mathrm{V}]}{dt} &= q_{-1}[\mathrm{V}^*] - q_1[\mathrm{V}] + q_7[\mathrm{mVVD}] - q_3[\mathrm{V}] + 2l_9[\mathrm{V}^*\mathrm{V}^*] + l_4[\mathrm{W}^*\mathrm{V}^*] + l_6[\mathrm{W_p^*}\mathrm{V}^*], \\
\frac{d[\mathrm{V}^*]}{dt} &= -q_{-1}[\mathrm{V}^*] + q_1[\mathrm{V}] - 2q_2[\mathrm{V}^*]^2 + 2q_{-2}[\mathrm{V}^*\mathrm{V}^*] - l_1[\mathrm{W}^*][\mathrm{V}^*] + l_{-1}[\mathrm{W}^*\mathrm{V}^*] - l_1[\mathrm{W_p^*}][\mathrm{V}^*] + l_{-1}[\mathrm{W_p^*}\mathrm{V}^*] \\
&+ l_5[\mathrm{W}^*\mathrm{V}^*] + l_7[\mathrm{W_p^*}\mathrm{V}^*] - q_4[\mathrm{V}^*], \\
\frac{d[\mathrm{V}^*\mathrm{V}^*]}{dt} &= q_2[\mathrm{V}^*]^2 - q_{-2}[\mathrm{V}^*\mathrm{V}^*] - l_9[\mathrm{V}^*\mathrm{V}^*] - q_5[\mathrm{V}^*\mathrm{V}^*], \\
\frac{d[\mathrm{W}^*\mathrm{V}^*]}{dt} &= l_1[\mathrm{W}^*][\mathrm{V}^*] - l_{-1}[\mathrm{W}^*\mathrm{V}^*] - l_2[\mathrm{W}^*\mathrm{V}^*] - k_5[\mathrm{W}^*\mathrm{V}^*]\frac{[\mathrm{FRQ}]^{n_5}}{([\mathrm{FRQ}]^{n_5} + K_5^{n_5})} + k_{-5}[\mathrm{W_p^*}\mathrm{V}^*] \\
&- (l_4 + l_5)[\mathrm{W}^*\mathrm{V}^*], \\
\frac{d[\mathrm{W_p^*}\mathrm{V}^*]}{dt} &= l_1[\mathrm{W_p^*}][\mathrm{V}^*] - l_{-1}[\mathrm{W_p^*}\mathrm{V}^*] - l_3[\mathrm{W_p^*}\mathrm{V}^*] + k_5[\mathrm{W}^*\mathrm{V}^*]\frac{[\mathrm{FRQ}]^{n_5}}{([\mathrm{FRQ}]^{n_5} + K_5^{n_5})} - k_{-5}[\mathrm{W_p^*}\mathrm{V}^*] \\
&- (l_6 + l_7)[\mathrm{W_p^*}\mathrm{V}^*], \\
\frac{d[\mathrm{FRQ}]}{dt} &= k_{15}[\mathrm{mFRQ}] - k_{19}[\mathrm{FRQ}], \\
\frac{d[\mathrm{mWCC}]}{dt} &= \frac{k_{11}[\mathrm{W}^*\mathrm{W}^*]^{n_{11}}}{([\mathrm{W}^*\mathrm{W}^*]^{n_{11}} + K_{11}^{n_{11}})} + k_{12} - k_{17}[\mathrm{mWCC}], \\
\frac{d[\mathrm{mVVD}]}{dt} &= \frac{q_6[\mathrm{W}^*\mathrm{W}^*]^{n_6}}{([\mathrm{W}^*\mathrm{W}^*]^{n_6} + Q_6^{n_6})} - q_8[\mathrm{mVVD}], \\
\frac{d[\mathrm{mFRQ}]}{dt} &= \frac{k_{13}[\mathrm{W}^*\mathrm{W}^*]^{n_{13}}}{([\mathrm{W}^*\mathrm{W}^*]^{n_{13}} + K_{13}^{n_{13}})} + \frac{k_{14}[\mathrm{W}]^{n_{14}}}{([\mathrm{W}]^{n_{14}} + K_{14}^{n_{14}})} + \frac{k_{14}[\mathrm{W}^*\mathrm{W}^*]^{n_{14}}}{([\mathrm{W}^*\mathrm{W}^*]^{n_{14}} + K_{14'}^{n_{14}})} - k_{18}[\mathrm{mFRQ}].
\end{aligned}
$$

Parameter estimation

To estimate the parameters of the model, we fitted the light-induction data (Fig. 1) using two different methods: a Bayesian technique and a frequentist inference approach.

Bayesian inference and MCMC

In the Bayesian approach, the parameters are assumed to be random variables that follow a particular distribution. Inferences are made based on the posterior distribution of the parameters, given the data and prior information about the parameters.

We first construct the posterior probability distribution

$$p(q|d) \propto p(q)p(d|q), \tag{1}$$

where $p(\cdot|\cdot)$ denotes a conditional probability, d is the data and q the parameters to be determined. The left hand of Eq. (1) is the quantity in which we are interested.

The right hand side of Eq. (1) are quantities that we can calculate:

$p(q)$: The prior, $p(q)$ summarizes any prior information we have on the possible values of the parameters. Here we use an uninformative prior for each of the parameters.

$p(d|q)$: We assume that the error at each data point is Gaussian distributed to give

$$p(d|q) \propto \exp\left(\frac{|\tilde{d}_i - d_i|}{2\sigma^2}\right), \tag{2}$$

where the d_i are the experimental values at each ith time and $\tilde{d}_i$ are the estimated values obtained from the set of parameters, q.

A Markov chain with equilibrium distribution $p(q|d)$ is then generated using the Metropolis-Hastings algorithm (Metropolis *et al*, 1953; Hastings, 1970). New parameter values, q', are randomly drawn from the uniform distribution $U(q-\delta, q+\delta)$, where δ is the size of the random walk. The new candidate parameters are accepted with probability

$$\alpha = \min\{1, \frac{p(d|q')p(q')}{p(d|q)p(q)}\}.$$

If the sampling procedure converges for a particular parameter, then the parameter is well-determined by the data and statistics such as the mean and variance of the parameter distribution can be obtained.

We adopted a two-step procedure by first fitting the vvd^{SS692} light-induction data (Fig. 1). We fixed the fitted parameters and determined the remaining parameters involving VVD by fitting to the wild type data. A subset of parameters was fixed within the range of biochemically reasonable values. Hill coefficients were also restricted. The other parameters for which no such estimates were available were fitted. For our fits, 1×10^4 iterations were performed with a burn-in period of usually half the total number of iterations. Samples were collected every tenth iteration thereafter. Parameter values are listed in Tables S3-S7 with their standard deviations. These parameters have been used to simulate the results in the main paper.

Parameter		Description	Value	σ
k_{-1}		Photoadduct decay of W^*, W^*_p	0.17 hr^{-1}	*
k_2		W^* homodimerization	5093.08 hr^{-1} au^{-1}	115.66
k_{-2}		$W^*W^* \rightarrow W^* + W^*$ dissociation	3637.79 hr^{-1}	36.17
l_8		$W^*W^* \rightarrow W + W$ photoadduct decay	0.17 hr^{-1}	*
k_6		W degradation rate	0.0601 hr^{-1}	8.6 $\times 10^{-4}$
k_7		W^* degradation rate	0.15 hr^{-1}	0.0017
k_8		W^*W^* degradation rate	0.67 hr^{-1}	0.0091
k_{16}		W translation rate	0.9 hr^{-1}	–
K_{16}		Half-maximal rate W translation rate	0.20 au	0.0012
n_{16}		Hill coefficient W translation	0.96	0.015
k_{11}	(1)	Max. rate *wc-1* transcription	308 hr^{-1}	–
K_{11}	(1)	Half-maximal rate *wc-1* transcription	1.37 au	0.016
n_{11}	(1)	Hill coefficient *wc-1* transcription	0.97	0.0080
k_{12}		Basal *wc-1* transcription	13.49 hr^{-1}	0.19
k_{17}		*wc-1* degradation	8.2 hr^{-1}	–

Table S3: Transcription module parameters for WCC. (1) W*W*-driven transcription. * indicate parameters which were not fitted. – non identifiable parameters. Standard deviation σ are derived from the MCMC fitting.

Parameter		Description	Value	σ
k_{15}		FRQ translation rate	1.33 hr^{-1}	0.032
k_{19}		FRQ degradation rate	0.036 hr^{-1}	0.019
k_{13}	(1)	Max. rate *frq* transcription	250 hr^{-1}	–
K_{13}	(1)	Half-maximal rate *frq* transcription	0.09 a.u	–
n_{13}	(1)	Hill coefficient *frq* transcription	1.56	–
k_{14}	(1),(2)	Max.rate *frq* transcription	56.0 hr^{-1}	–
K_{14}	(2)	Half-maximal rate *frq* transcription	14.61 au	0.21
$K_{14'}$	(1)	Half-maximal rate *frq* transcription	0.0026 au	0.000016
n_{14}	(1),(2)	Hill coefficient *frq* transcription	1.92	–
k_{18}		*frq* degradation	9.8 hr^{-1}	

Table S4: Transcription module parameters for FRQ. (1) W*W*-driven transcription. (2) W-driven transcription. – non identifiable parameters.

Parameter		Description	Value	σ
q_{-1}		Photoadduct decay of V^*	0.17 hr^{-1}	*
q_2		V^* homodimerization	2732.45 hr^{-1} au^{-1}	69.80
q_{-2}		$V^*V^* \rightarrow V^* + V^*$ dissociation	3034.19 hr^{-1}	73.74
l_9		$V^*V^* \rightarrow V + V$ photoadduct decay	0.17 hr^{-1}	*
q_3		V degradation rate	0.63 hr^{-1}	0.058
q_4		V^* degradation rate	0.60 hr^{-1}	0.031
q_5		V^*V^* degradation rate	1.41 hr^{-1}	0.022
q_7		V translation rate	12.36 hr^{-1}	0.39
q_8		*vvd* degradation rate	6.40 hr^{-1}	–
q_6	(1)	Max.rate *vvd* transcription	1595 hr^{-1}	–
Q_6	(1)	Half-maximal rate *vvd* transcription	0.42 au	–
n_6	(1)	Hill coefficient *vvd* transcription	1.4	–

Table S5: Transcription module parameters for VVD. * indicate parameters which were not fitted. – non identifiable parameters. (1) W*W*-driven transcription.

Parameter	Description	Value	σ
l_1	heterodimerization	1.95 hr^{-1} au^{-1}	0.050
l_{-1}	heterodimer dissociation	0.016 hr^{-1}	0.0022
l_2	W^*V^* degradation	0.057 hr^{-1}	0.0016
l_3	$W_p^*V^*$ degradation	0.023 hr^{-1}	0.0056
l_4	$W^*V^* \rightarrow W^* + V$	0.02 hr^{-1}	–
l_5	$W^*V^* \rightarrow W + V^*$	0.8 hr^{-1}	*
l_6	$W_p^*V^* \rightarrow W_p^* + V$	0.15 hr^{-1}	0.0024
l_7	$W_p^*V^* \rightarrow W_p + V^*$	0.09 hr^{-1}	–

Table S6: Heterodimerization and photoadduct decay module parameters. – non identifiable parameters.

Parameter	Description	Value	σ
k_3	Max. rate W phosphorylation	1.89 hr^{-1}	–
K_3	Half-maximal rate W phosphorylation	12.34 au	0.13
k_{-3}	W_p dephosphorylation rate W	1.26 hr^{-1}	0.015
n_3	Hill coefficient W phosphorylation	1.0	–
k_4	Max. rate W^* phosphorylation	20.69 hr^{-1}	–
K_4	Half-maximal rate W^* phosphorylation	177.08 hr^{-1}	7.73
k_{-4}	W_p^* dephosphorylation rate	0.52 hr^{-1}	0.016
n_4	Hill coefficient W^* phosphorylation	1.78	0.070
k_5	Max.rate W^*V^* phosphorylation	0.16 hr^{-1}	–
K_5	Half-maximal rate W^*V^* phosphorylation	10.73 au	0.23
k_{-5}	$W_p^*V^*$ dephosphorylation rate	1.15 hr^{-1}	0.70
n_5	Hill coefficient W^*V^* phosphorylation	1	–
k_9	W_p degradation	0.16 hr^{-1}	–
k_{10}	W_p^* degradation	0.15 hr^{-1}	–

Table S7: Phosphorylation module parameters. – non identifiable parameters.

Maximum likelihood estimation and profile likelihood analysis

A more traditional approach to parameter fitting is that of maximum likelihood estimation. Here, one first describes an objective function which defines the agreement of the experimental data with the predictions of the model. Commonly, this is the weighted sum of squared residuals:

$$\chi^2(\theta) \quad = \quad \sum_{k=1}^{m}\sum_{l=1}^{d}\left(\frac{y_{kl}^D - y_k(\theta, t_l)}{\sigma_{kl}^D}\right)^2,$$

where y_{kl}^D denotes d-data points for each variable k at time points t_l. The σ_{kl}^D are the corresponding measurement errors. The model predictions for a set of parameters θ are given by $y_k(\theta, t_l)$. The parameters are then estimated by

$$\hat{\theta} \quad = \quad \min_{\theta}[\chi^2(\theta)],$$

giving a point estimate of the parameters that give the best fit of the model to the data. For normally distributed noise, this is also the negative log likelihood of the data being observed for a given set of parameters θ:

$$\chi^2(\theta) \quad = \quad \text{constant} \; - 2\log(\mathrm{L}(\theta).$$

Given that there are usually a large number of parameters that are to be determined by a much smaller number of experiments, it is important to infer how well the parameters can be identified. The problem of identifiability can be addressed by use of the profile likelihood which can be used to distinguish between structural and practically non-identifiable parameters as well as calculating confidence intervals for the parameters (Raue *et al*, 2009, 2011; Steiert *et al*, 2012). The profile likelihood is calculated for each parameter by

$$\chi^2_{PL}(\theta) \quad = \quad \min_{\theta_{j \neq i}}[\chi^2(\theta)],$$

which means $\chi^2(\theta)$ is re-optimized with respect to all remaining parameters $\theta_{j\neq i}$ for each parameter θ_i in turn. The shape of the profile likelihood then distinguishes between the different types of identifiability.

A threshold, Δ_α can be derived from the log-likelihood of the best fit and the α quantile of the χ^2 distribution to define a confidence interval. A confidence interval of parameter θ_i, $[\sigma_i^-, \sigma_i^+]$, to a confidence level α implies that the true value of θ_i lies within this interval with probability α. A parameter is defined as identifiable if the confidence interval $[\sigma_i^-, \sigma_i^+]$ is finite. For a parameter which is identifiable, the χ^2 stays below the threshold Δ_α for a desired confidence level α but then exceeds this threshold at finite values (Raue *et al*, 2009). If $\chi^2(\theta)$ is constant then the parameter is said to exhibit structural non-identifiabiity and its value cannot be determined. Practical non-identifiability occurs if, in one direction, the increase in $\chi^2(\theta)$ stays below the threshold Δ_α (Raue *et al*, 2009). In this case, the confidence interval of the parameter can be finitely bounded in one direction and in the other, it is infinite. An algorithm that can be used as a starting point for calculating the profile likelihood is given in Raue *et al* (2009). Using this framework, we re-fitted the light-induction data (Fig S4).

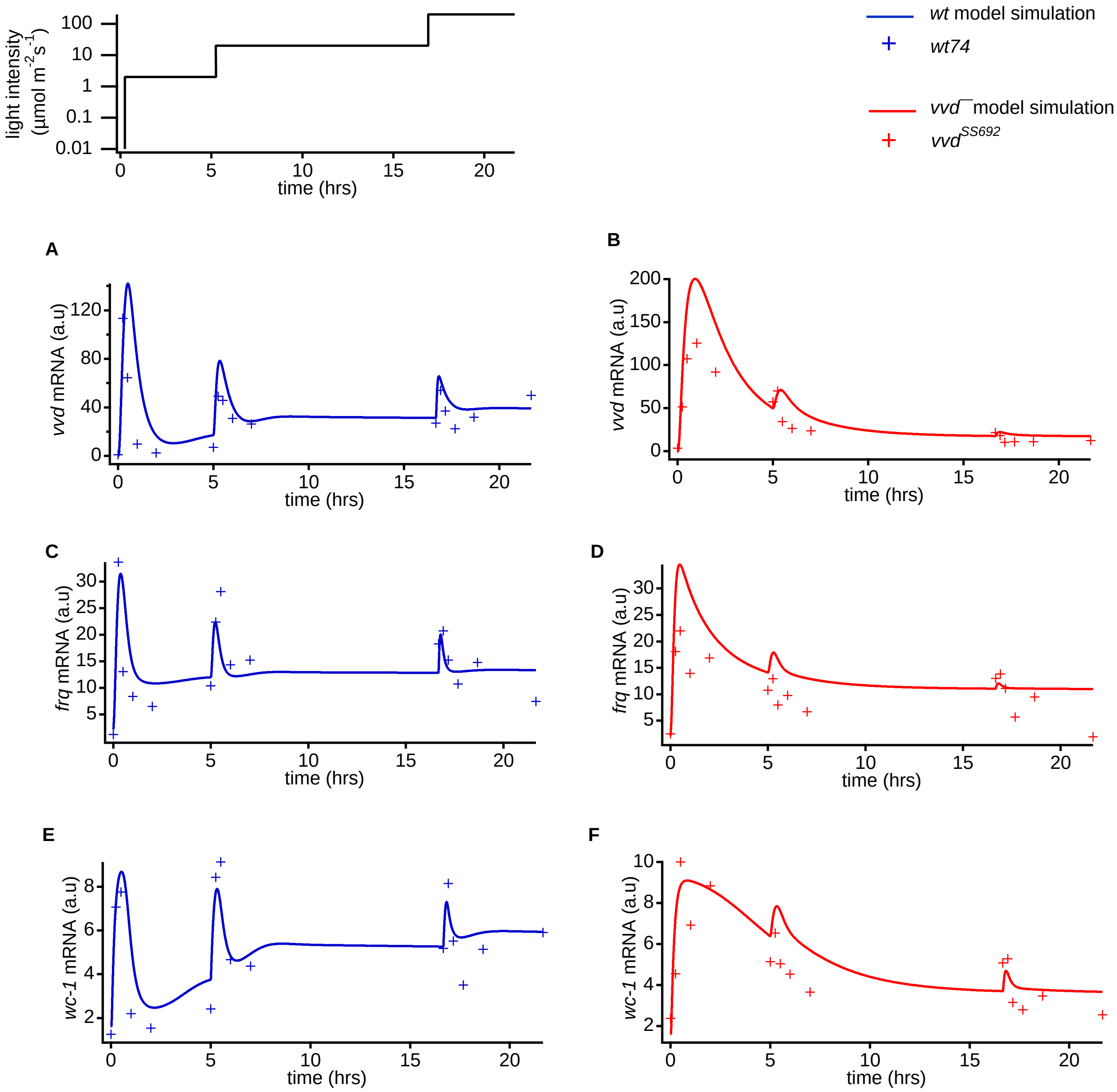

Figure S4: **Fitting of the light adaptation data using a maximum likelihood optimization approach** Fitting shows that the key features of the experimental results can be reproduced (blue curves: wild type; red curves: *vvd-* mutant). The wild type maintains its responsiveness to the subsequent increases in light intensity, while the successive peaks of the mRNA levels decrease in the *vvd-* mutant.

The profile likelihoods for the parameters related to VVD (q_6, q_7, q_8), heterodimerization $(l_1,\ l_{-1})$ and the rate of replenishment of the inactive WCC pool, l_5 are shown in Fig. S5. The dashed red line indicates the confidence interval for a confidence level of $\alpha = 0.95$. The parameters could not be fully identified, although upper and lower bounds could be obtained for some of the parameters. Parameter values of the best fit are listed with corresponding upper or lower limits on their values found from the profile likelihood analysis in the Tables S8-S12. The fitted values using the MCMC algorithm lie within these limits.

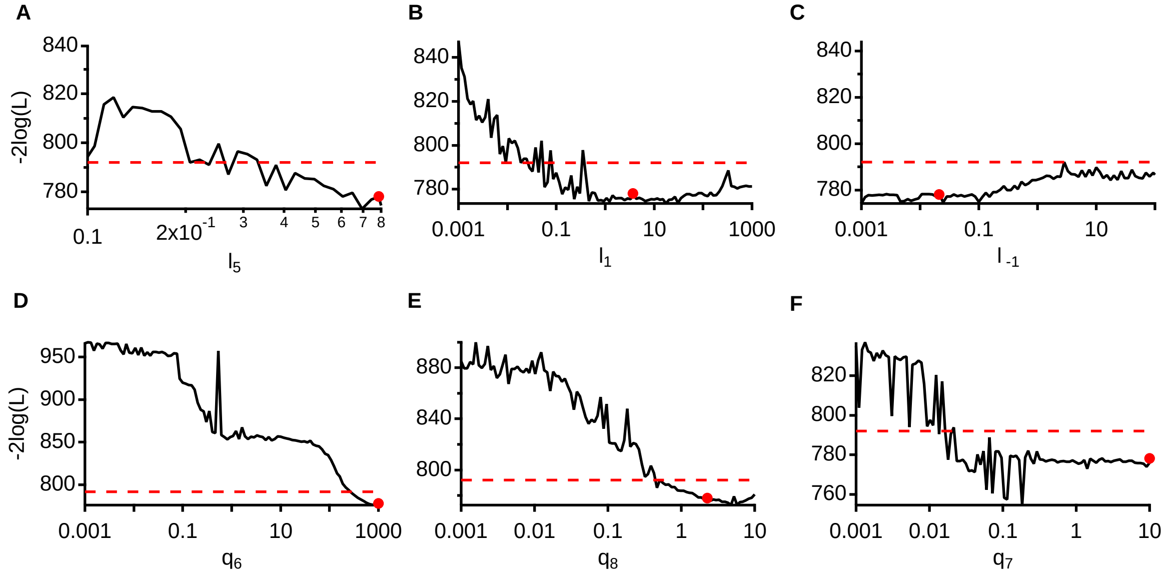

Figure S5: **Profile likelihoods for parameters fitted using the method of Raue et al.** The profile likelihoods for the parameters related to VVD (q_6, q_7, q_8), heterodimerization $(l_1,\ l_{-1})$ and the rate of replenishment of the inactive WCC pool, l_5. Red circles indicate the best fit. The dashed line indicates the 95% confidence intervals based on the χ^2 distribution; described in Raue *et al* (2009).

Of particular interest is that the rate of replenishment, l_5, is bounded below and the best fit is found at the upper range of the parameter space explored. The parameter space for l_5 as well as other photoadduct decay variables (l_4, l_6 and l_7) was truncated above at 0.8 hr^{-1}. This was done because better fits were found with increasing values of l_5, giving photoadduct decay times of less than an hour, while experimental measurements suggest that the rate of photoadduct decay occurs on the order of hours (Zoltowski *et al*, 2009).

Parameter		Description	Value	Range
k_{-1}		Photoadduct decay of W^*, W^*_p	0.17 hr^{-1}	–
k_2		W^* homodimerization	165.67 hr^{-1} au^{-1}	–
k_{-2}		$W^*W^* \rightarrow W^* + W^*$ dissociation	1478.11 hr^{-1}	–
l_8		$W^*W^* \rightarrow W + W$ photoadduct decay	0.17 hr^{-1}	*
k_6		W degradation rate	0.0635 hr^{-1}	–
k_7		W^* degradation rate	0.125 hr^{-1}	–
k_8		W^*W^* degradation rate	9.99 hr^{-1}	–
k_{16}		W translation rate	1.68 hr^{-1}	–
K_{16}		Half-maximal rate W translation rate	0.0011 au	<100
n_{16}		Hill coefficient W translation	4.56	–
k_{11}	(1)	Max. rate *wc-1* transcription	49.55 hr^{-1}	>10
K_{11}	(1)	Half-maximal rate *wc-1* transcription	0.060 au	
n_{11}	(1)	Hill coefficient *wc-1* transcription	2.4	–
k_{12}		Basal *wc-1* transcription	10.31 hr^{-1}	<5
k_{17}		*wc-1* degradation	6.42 hr^{-1}	>1

Table S8: Transcription module parameters for WCC. (1) W*W*-driven transcription. * indicate parameters which were not fitted. – non identifiable parameters.

Parameter		Description	Value	Range
k_{15}		FRQ translation rate	1.35 hr^{-1}	–
k_{19}		FRQ degradation rate	0.32 hr^{-1}	–
k_{13}	(1)	Max. rate *frq* transcription	329.34 hr^{-1}	–
K_{13}	(1)	Half-maximal rate *frq* transcription	0.29 a.u	–
n_{13}	(1)	Hill coefficient *frq* transcription	1.5	–
k_{14}	(1),(2)	Max.rate *frq* transcription	68.35 hr^{-1}	>5
K_{14}	(2)	Half-maximal rate *frq* transcription	78.20 au	–
$K_{14'}$	(1)	Half-maximal rate *frq* transcription	0.0010 au	–
n_{14}	(1),(2)	Hill coefficient *frq* transcription	1.00	–
k_{18}		*frq* degradation	7.44 hr^{-1}	>1

Table S9: Transcription module parameters for FRQ. (1) W*W*-driven transcription. (2) W-driven transcription. – non identifiable parameters.

Parameter		Description	Value	Range
q_{-1}		Photoadduct decay of V^*	0.17 hr^{-1}	*
q_2		V^* homodimerization	1343.21 hr^{-1} au^{-1}	–
q_{-2}		$V^*V^* \rightarrow V^* + V^*$ dissociation	100.04 hr^{-1}	–
l_9		$V^*V^* \rightarrow V + V$ photoadduct decay	0.17 hr^{-1}	*
q_3		V degradation rate	0.10 hr^{-1}	–
q_4		V^* degradation rate	0.20 hr^{-1}	–
q_5		V^*V^* degradation rate	1.13 hr^{-1}	–
q_7		V translation rate	9.97 hr^{-1}	>0.02
q_8		*vvd* degradation rate	2.26 hr^{-1}	>0.6
q_6	(1)	Max.rate *vvd* transcription	997.59 hr^{-1}	>300
Q_6	(1)	Half-maximal rate *vvd* transcription	0.27 au	$0.02, 60$
n_6	(1)	Hill coefficient *vvd* transcription	1.7	<3

Table S10: Transcription module parameters for VVD. * indicate parameters which were not fitted. – non identifiable parameters. (1) W*W*-driven transcription.

Parameter	Description	Value	Range
l_1	heterodimerization	3.67 hr^{-1} au^{-1}	>0.5
l_{-1}	heterodimer dissociation	0.021 hr^{-1}	–
l_2	W^*V^* degradation	0.01 hr^{-1}	<0.2
l_3	$W_p^*V^*$ degradation	0.01 hr^{-1}	<2
l_4	$W^*V^* \rightarrow W^* + V$	0.049 hr^{-1}	–
l_5	$W^*V^* \rightarrow W + V^*$	0.78 hr^{-1}	>0.2
l_6	$W_p^*V^* \rightarrow W_p^* + V$	0.001 hr^{-1}	–
l_7	$W_p^*V^* \rightarrow W_p + V^*$	0.79 hr^{-1}	–

Table S11: Heterodimerization and photoadduct decay module parameters. – non identifiable parameters.

Parameter	Description	Value	Range
k_3	Max. rate W phosphorylation	0.64 hr^{-1}	–
K_3	Half-maximal rate W phosphorylation	7.25 au	–
k_{-3}	W_p dephosphorylation rate W	3.11 hr^{-1}	–
n_3	Hill coefficient W phosphorylation	5	–
k_4	Max. rate W^* phosphorylation	9.71 hr^{-1}	–
K_4	Half-maximal rate W^* phosphorylation	8.35 hr^{-1}	–
k_{-4}	W_p^* dephosphorylation rate	0.84 hr^{-1}	–
n_4	Hill coefficient W^* phosphorylation	3.92	–
k_5	Max.rate W^*V^* phosphorylation	0.016 hr^{-1}	–
K_5	Half-maximal rate W^*V^* phosphorylation	266.10 au	–
k_{-5}	$W_p^*V^*$ dephosphorylation rate	6.11 hr^{-1}	–
n_5	Hill coefficient W^*V^* phosphorylation	1	–
k_9	W_p degradation	0.032 hr^{-1}	–
k_{10}	W_p^* degradation	0.14 hr^{-1}	<0.8

Table S12: Phosphorylation module parameters. – non identifiable parameters.

Supplementary Text 2

Futile cycling provides the means for repeated induction

In order to demonstrate that futile cycling provides a mechanism that could account for repeated sensitivity to increasing step changes in stimuli levels, we constructed a simplified model that represents the core features of adaptation in the *Neurospora* model (Fig. S6A). Here the inactive species X is activated by an external stimulus to give X*, the activated species which in turn synthesizes its inhibitor I. The activated species is able to form a complex with the the inhibitor. This complex C, (corresponding to the WCC-VVD heterodimer in Neurospora) is able to either dissociate to its two components or dissociate with the activated component X* reverting to its inactivated state, X. The parameter k_{repl} describes the rate of replenishment. We first fitted the *vvd* mRNA data to this model and showed that this reduced model contains the elements necessary to reproduce the wild type and *vvd*- mutant behavior: adaptation is achieved in both the wild type and mutant case, with the mutant peaks decreasing in increasing stimulus levels (Fig. S6).

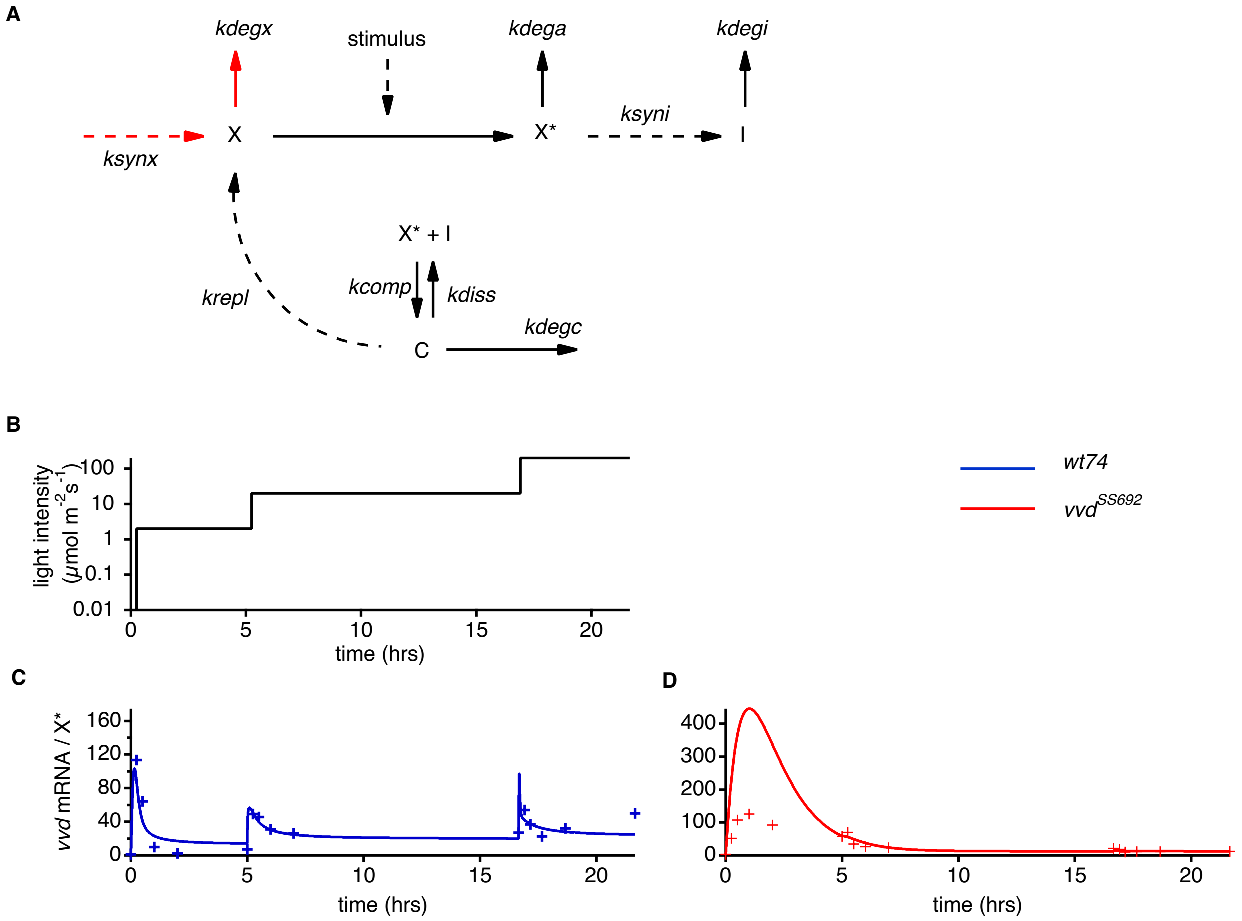

Figure S6: **Futile cycling mediates repeated responsiveness.**
(A): Simplified network to highlight the role of futile cycling. Upon stimulation, X is activated to state X*, which then synthesizes the inhibiting species, I. X* and I interact to form the complex C. C can reversibly dissociate but can also dissociate such that the active X* reverts back to its inactive form X. This constitutes the futile cycling component. The parameters k_{synx} and k_{degx} (marked red) are fixed at 10 a.u hr $^{-1}$ and 0.01 hr^{-1} respectively. (B)-(D): The key features of adaptation and the differences between the wild type and the *vvd*- mutant are reproduced by the reduced model.

We performed a profile likelihood analysis and found that similar to fitting the full *Neurospora* model, not all parameters can be uniquely determined. However, in contrast, the parameter values are restricted

either on an upper or a lower boundary (Fig. S7). Of note is that the parameter crucial to the futile cycle, k_{repl}, is clearly identifiable (Fig. S7A). If the value k_{repl} lies to the right of the confidence interval, then the replenishment rate is too fast and the replenished X is reactivated and therefore, effective adaptation cannot be achieved. On the other hand, if it is too slow, then the wild type tends to the *vvd-* mutant behavior with diminishing responses to increased stimuli levels.

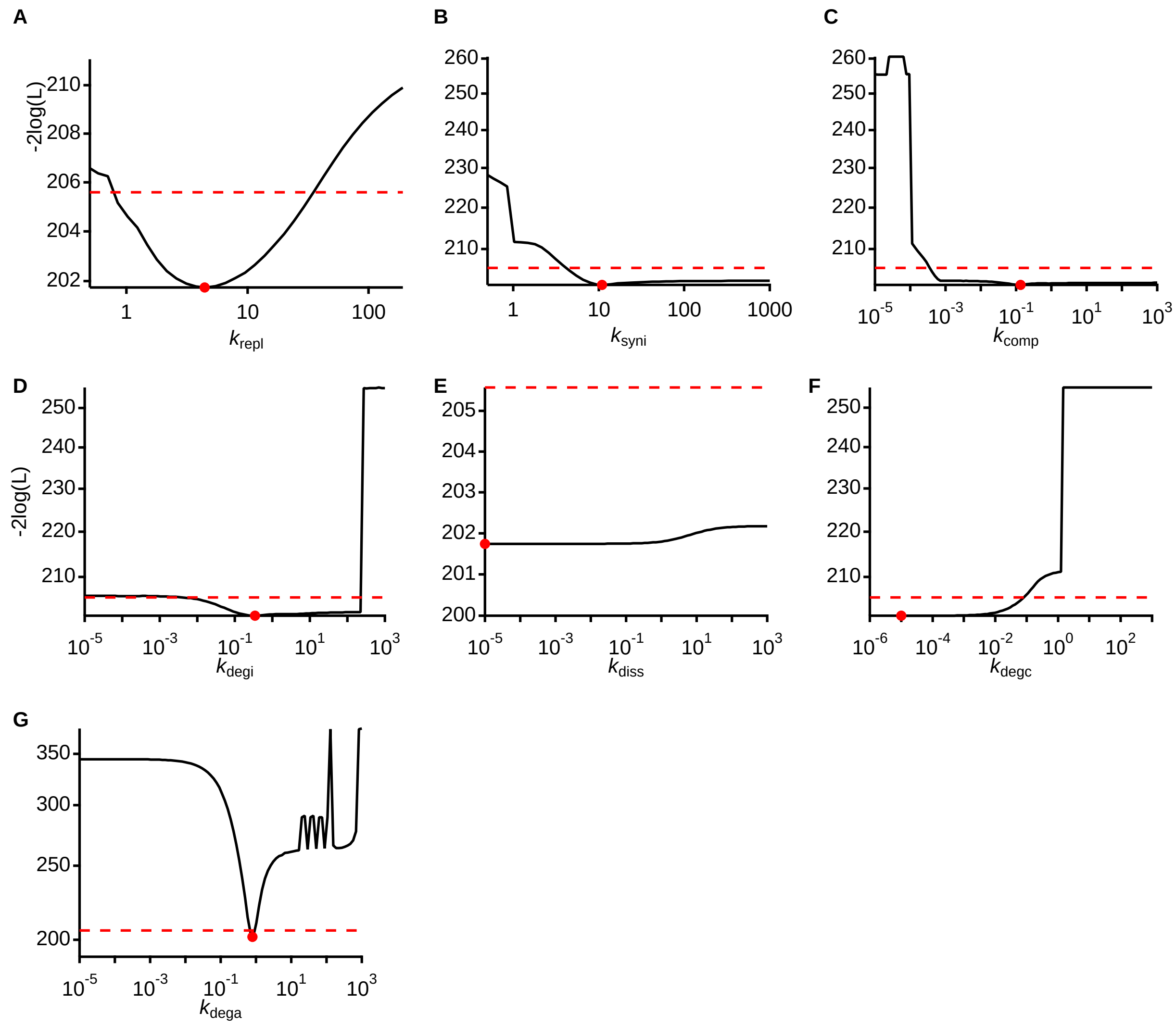

Figure S7: **Profile likelihood for parameters of the reduced model, fitted to the experimental *vvd* mRNA data.**
Red circles indicate the best fit. The dashed line indicates the 95% confidence interval based on the χ^2 distribution. Parameters (A) - (F) belong to the futile cycle part of the model corresponding to the synthesis and degradation of the inhibitor, I, complex formation and dissociation, and replenishment of the inactive species. Degradation of the activated state X* is shown in (G).

To generalize the result that futile cycling is a method that can be used to achieve repeated responsiveness, we optimized the parameters of the model (Fig. S6) by defining an objective function expressing the qualitative features of adaptation, rather than fitting directly to data. The characteristics of the peak of the responses of a wild type and a mutant system were used to place constraints on the parameters, giving us a constrained optimization problem.

The two key features of adaptation - the ability of a system to respond to step inputs and then to return to the pre stimulus level in the continued presence of the stimulus - can be defined as the sensitivity and precision of a network (Ma *et al*, 2009). The features that are required to determine the sensitivity and precision are the peak height P_i, the adapted level, O_i and the input levels, I_i (Fig. S8).

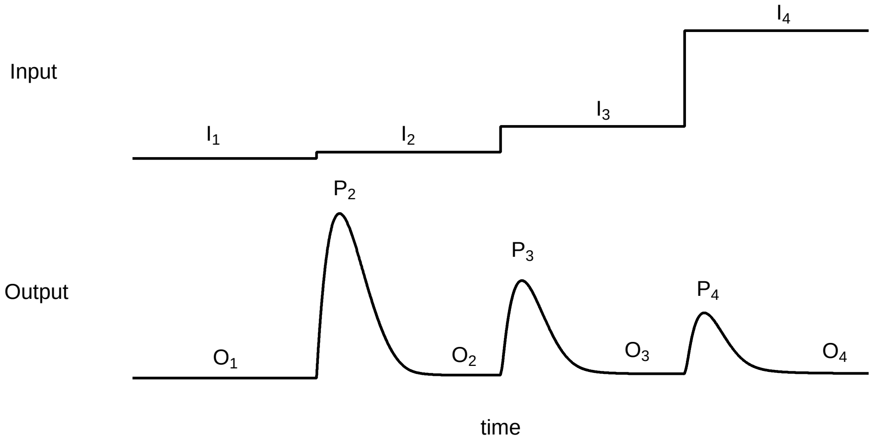

Figure S8: **Adaptation and repeated sensitivity.**
An input I is stepped up successively and at each step increase, the system should respond giving a significant peak, P_i, but then return to its pre-stimulus level, O_i in the continued presence of the input.

Sensitivity is the relative change in the system peak (P_2) compared to the steady-state value before the stimulus (O_1) with reference to the change in input levels I_1 and I_2:

$$\left| \frac{(P_2 - O_1)/O_1}{(I_2 - I_1)/I_1} \right| \tag{3}$$

Precision is given by the difference between the pre-stimulus and post-stimulus steady-state values (defined as the inverse of the relative error by Ma *et al* (2009)):

$$\left| \frac{(O_2 - O_1)/O_1}{(I_2 - I_1)/I_1} \right|^{-1} \tag{4}$$

In contrast to other studies of adaptation, we are interested in the repeated response of a system to further step increases in the stimulus level. Therefore we phrase the sensitivity and precision with respect to $n = 4$ increasing stimulus levels (Fig. S8). For the objective function, we used the definition of precision:

$$P = \sum_{i=2}^{n} \left| \frac{(O_i - O_1)/O_1}{(I_i - I_1)/I_1} \right|^{-1}, \tag{5}$$

where the precision is defined with respect to the pre-stimulus steady-state value, O_1.

A key difference between the wild type and the *vvd-* mutant lies in the magnitude of the responses elicited at each successive, increasing stimulus level. We utilize this fact to define the following constraints to the optimization problem. In the case of the wild type, successive peaks heights should not be diminished:

$$\frac{P_3}{P_2} \geq P_{\text{wildtype}}, \tag{6}$$

$$\frac{P_4}{P_2} \geq P_{\text{wildtype}},$$

where we set $P_{\text{wildtype}} = 0.8$.

In the case of the mutant behavior, the system should no longer be sensitive and the successive peak heights should decrease:

$$\frac{P_3}{P_2} \leq P_{\text{mutant}}, \qquad (7)$$

$$\frac{P_4}{P_2} \leq P_{\text{mutant}},$$

where $P_{\text{mutant}} = 0.1$. For both constraints we specify P_3 and P_4 with respect to the first peak, P_2.

We used the Matlab function `fmincon` with the interior-point algorithm to determine the parameters that solves:

$$\min_q F(q) \quad = \quad \sum_{i=2}^{n} \left| \frac{(O_i - O_1)/O_1}{(I_i - I_1)/I_1} \right|. \qquad (8)$$

subject to the constraints (7) and (8) for the network shown in Fig. S6, where F is the relative error (Eq. (4)) The input levels are: $I_1 = 10^{-6}$; $I_2 = 1$; $I_3 = 10$ and $I_4 = 100$. The optimization was run for 300 sets of randomly-selected initial parameter values in the interval $[10^{-4}\ 10]$.

Similar to constructing a profile likelihood by recalculation of the χ^2, we also investigated each parameter in turn, starting from the parameter set of the best fit and re-optimizing the system subject to the constraints. While fitting to data provides a natural measure of a good fit (for example, the χ^2), here we utilize the fact that the constraints, defining a wild type and *vvd*- mutant network, must be met and thus sets the criteria for a successful fit. The best fit of the 300 parameter estimation runs is marked by a red circle, while the dashed, vertical lines indicate the boundary at which the constraints are no longer met (Fig. S9). Panels A-C show the parameters related to the futile cycle motif: replenishment of X, and the formation and dissociation of the complex C. While there is no significant change in the minimum value over the range of the parameter search, the rate of replenishment k_{repl}, is bounded below giving a critical value where the constraints are no longer met. The same is also true of the synthesis rate of the inhibitor I, k_{syni} (D). The degradation rate of C, k_{degc} (F), is also a factor in ensuring the constraints are met, as is the degradation of the activated species, X* (G). The constraints specifically distinguish between a wild type and a mutant situation. The boundaries of these constraints show that these parameters must be non-zero, and support the idea that the futile cycling motif is capable of producing adaptation and maintaining responsiveness.

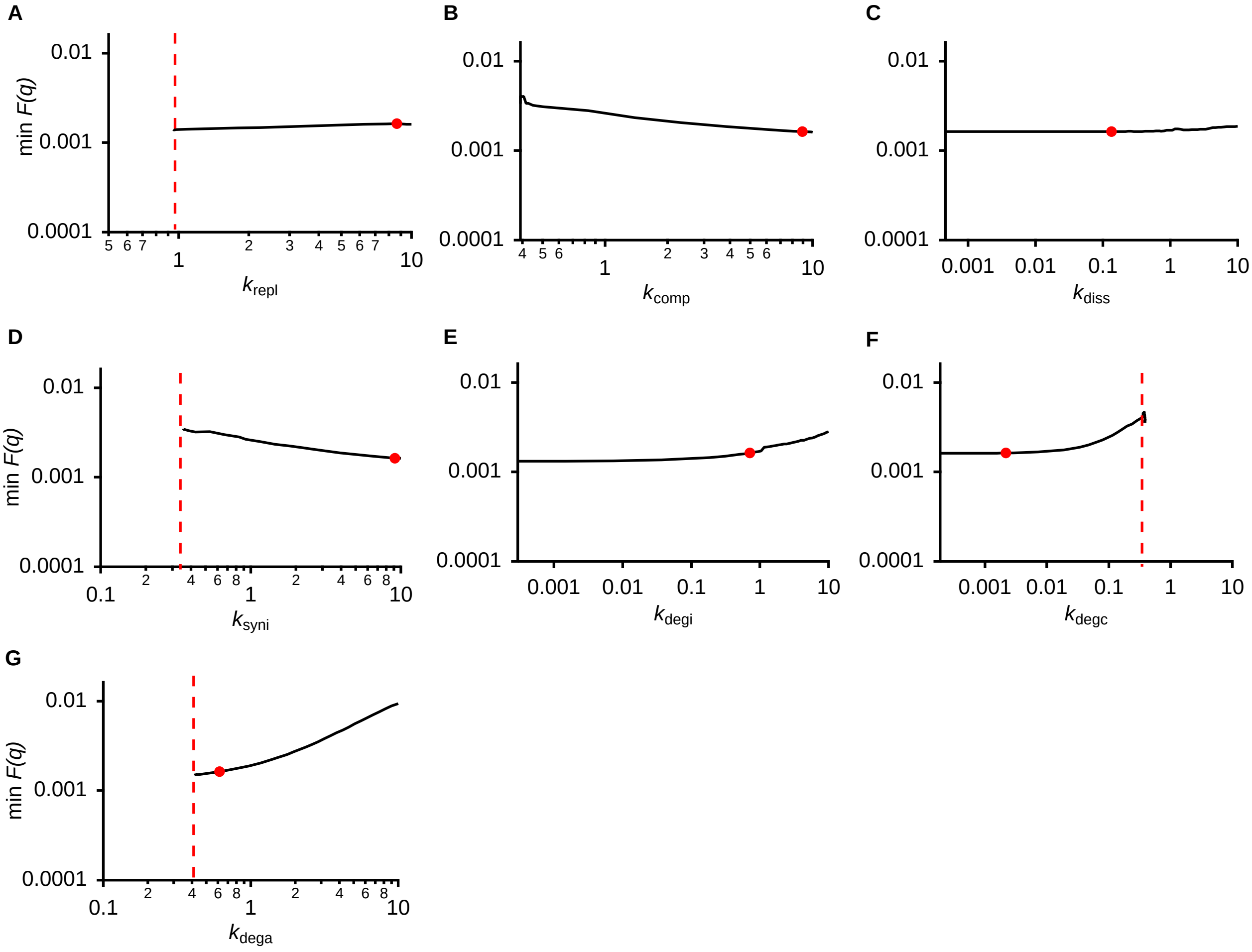

Figure S9: **Defined constraints on the peak heights specify bounds on the parameter values.**
(A) - (C): Parameters for replenishment of the pool of X, complex formation and complex dissociation ('wild type' mode parameters). Red circles mark the best fit. The dashed red lines indicate the threshold at which the constraints are no longer satisfied.
(D) - (F): Parameters describing the synthesis and degradation of the inhibitor I and the rate of complex degradation.
(G): Degradation rate of the activated species X*.

References

Baker C, Loros J, Dunlap J (2012) The circadian clock of Neurospora crassa. *FEMS Microbiol Rev* **36**: 95–110

Brunner M, Káldi K (2008) Interlocked feedback loops of the circadian clock of *Neurospora crassa*. *Molecular Microbiology* **68**: 255–262

Chen C, DeMay BS, Gladfelter AS, Dunlap JC, Loros JJ (2010) Physical interaction between VIVID and white collar complex regulates photoadaptation in *Neurospora*. *PNAS* **107**: 16715–16720

Elvin M, Loros JJ, Dunlap JC, Heintzen C (2005) The PAS/LOV protein VIVID supports a rapidly dampened daytime oscillator that facilitates entrainment of the *Neurospora* circadian clock. *Genes Development* **19**: 2593–2605

Froehlich AC, Loros JJ, Dunlap JC (2003) Rhythmic binding of a WHITE COLLAR-containing complex to the frequency promoter is inhibited by FREQUENCY. *PNAS* **100**: 5914–5919

Hastings W (1970) Monte Carlo sampling methods using Markov chains and their applications. *Biometrika* **57**: 97–109

Heintzen C, Loros JJ, Dunlap JC (2001) The PAS Protein VIVID Defines a Clock-Associated Feedback Loop that Represses Light Input, Modulates Gating, and Regulates Clock Resetting. *Cell* **104**: 453–464

Hunt SM, Elvin M, Crosthwaite SK, Heintzen C (2007) The PAS/LOV protein VIVID controls temperature compensation of circadian clock phase and development in *Neurospora crassa*. *Genes Development* **21**: 1964–1974

Ma W, Trusina A, El-Samad H, Lim WA, Tang C (2009) Defining Network Topologies that Can Achieve Biochemical Adaptation. *Cell* **138**: 760–773

Malzahn E, Ciprianidis S, Káldi K, Schafmeier T, Brunner. M (2010) Photoadaptation in *Neursopora* by Competitive Interaction of Activating and Inhibitory LOV domains. *Cell* **142**: 762–772

Metropolis N, Rosenbluth A, Rosenbluth M, Teller A, Teller E (1953) Equations of state calculations by fast computing machines. *J Chem Phys* **21**: 1087–1092

Raue A, Kreutz C, Maiwald T, Bachmann J, Schilling M, Klingmüller U, Timmer J (2009) Structural and practical identifiability analysis of partially observed dynamical models by exploiting the profile likelihood. *Bioinformatics* **25**: 1923–1929

Raue A, Kreutz C, Maiwald T, Klingmüller U, Timmer J (2011) Addressing parameter identifiability by model-based experimentation. *IET Syst Biol* **5**: 120–130

Schafmeier T, Haase A, Káldi K, Scholz J, Fuchs M, Brunner M (2005) Transcriptional Feedback of *Neurospora* Circadian Clock Gene by phosphorylation-dependent Inactivation of Its Transcription Factor. *Cell* **122**: 235–246

Schafmeier T, Káldi K, Diernfellner A, Mohr C, Brunner M (2006) Phosphorylation-dependent maturation of *Neurospora* circadian clock protein from a nuclear repressor towards a cytoplasmic activator. *Genes Development* **20**: 297–306

Steiert B, Raue A, Timmer J, Kreutz C (2012) Experimental Design for Parameter Estimation of Gene Regulatory Networks. *PLos ONE* **7**: e40052

Zoltowski B, Crane B (2008) Light activation of the LOV protein Vivid generates a rapidly exchanging dimer. *Biochemistry* **47**: 7012–7019

Zoltowski B, Vaccaro B, Crane B (2009) Mechanism-based tuning of a LOV domain photoreceptor. *Nat Chem Biol* **5**: 827–834
